# TrpNet: Understanding Tryptophan Metabolism across Gut Microbiome

**DOI:** 10.3390/metabo12010010

**Published:** 2021-12-23

**Authors:** Yao Lu, Jasmine Chong, Shiqian Shen, Joey-Bahige Chammas, Lorraine Chalifour, Jianguo Xia

**Affiliations:** 1 Department of Microbiology and Immunology, McGill University, Montreal, QC H3A 2T5, Canada; yao.lu5@mail.mcgill.ca; 2 Institute of Parasitology, McGill University, Montreal, QC H3A 2T5, Canada; jasmine.chong@mail.mcgill.ca; 3 MGH Center for Translational Pain Research, Massachusetts General Hospital, Harvard Medical School, Boston, MA 02215, USA; SSHEN2@mgh.harvard.edu; 4 Department of Medicine, McGill University, Montreal, QC H3A 2T5, Canada; joey-bahige.chammas@mail.mcgill.ca (J.-B.C.); lorraine.chalifour@mcgill.ca (L.C.); 5 Lady Davis Institute for Medical Research, Montreal, QC H3T 1E2, Canada

**Keywords:** tryptophan metabolism, gut microbiome, co-metabolism, genome-scale metabolic model, network, indole derivatives

## Abstract

Crosstalk between the gut microbiome and the host plays an important role in animal development and health. Small compounds are key mediators in this host–gut microbiome dialogue. For instance, tryptophan metabolites, generated by biotransformation of tryptophan through complex host–microbiome co-metabolism can trigger immune, metabolic, and neuronal effects at local and distant sites. However, the origin of tryptophan metabolites and the underlying tryptophan metabolic pathway(s) are not well characterized in the current literature. A large number of the microbial contributors of tryptophan metabolism remain unknown, and there is a growing interest in predicting tryptophan metabolites for a given microbiome. Here, we introduce TrpNet, a comprehensive database and analytics platform dedicated to tryptophan metabolism within the context of host (human and mouse) and gut microbiome interactions. TrpNet contains data on tryptophan metabolism involving 130 reactions, 108 metabolites and 91 enzymes across 1246 human gut bacterial species and 88 mouse gut bacterial species. Users can browse, search, and highlight the tryptophan metabolic pathway, as well as predict tryptophan metabolites on the basis of a given taxonomy profile using a Bayesian logistic regression model. We validated our approach using two gut microbiome metabolomics studies and demonstrated that TrpNet was able to better predict alterations in in indole derivatives compared to other established methods.

## 1. Introduction

The gut microbiome is a community of metabolically active microorganisms inhabiting all niches along the intestines that coevolves with its host. Growing evidence has shown that the gut microbiome plays a critical role in animal development and health [1]. Disruptions in microbiome composition, termed dysbiosis, are implicated in various diseases including gastrointestinal diseases [2], infectious diseases [3], metabolic diseases [4,5], and neurological disorders [6]. Dysbiosis leads to a shift in the production of various microbial metabolites which then influence the physiology and immune status of the host [7]. Among these bioactive metabolites, short-chain fatty acids (SCFAs, produced by bacteria from fermenting dietary fibers), secondary bile acids (originated in liver and transformed by gut microbiome), and tryptophan-derived metabolites are most well known.

Microbes can degrade tryptophan to a range of indoles including indolelactate (ILA), indoleacetic acid (IAA), indolealdehyde (IAld), indoleacrylic acid (IA), and indolepropionate (IPA). These can activate the aryl hydrocarbon receptor (AhR), a transcription factor widely expressed by cells in the immune system, regulate intestinal homeostasis [8], initiate an immune response [9], and control oxidative stress defense [10]. AhR activation is associated with multiple diseases such as inflammatory bowel disease (IBD) [11], type 2 diabetes [12], and central nervous system (CNS)-related disorders [13].

To understand the health impact of tryptophan metabolites in host–microbiome interactions, it is essential to have detailed knowledge of tryptophan metabolism. Researchers can find related reactions and enzymes in several databases including Kyoto Encyclopedia of Genes and Genomes (KEGG) [14], BioCyc [15], Small Molecule Pathway Database (SMPDB) [16], and WikiPathways [17]. Among them, SMPDB and WikiPathways are mainly concerned with host tryptophan metabolism, while KEGG and BioCyc provide very limited information on tryptophan degradation across the gut microbiome. In particular, the origins of tryptophan metabolism are assigned at the enzyme level, and it is difficult to obtain the reactions and metabolites of a single species or from the microbial community. Moreover, not all tryptophan metabolites can be found in these databases. For instance, IPA, an important neuroprotective antioxidant produced by the human gut microbe represented by *Clostridium sporogenes*, is currently missing in KEGG.

Predicting metagenomic functions on the basis of microbiome composition has attracted great attention in recent years. Current tools, such as PICRUSt [18] and Tax4Fun [19], focus on enzyme- and pathway-level predictions and cannot be directly used to understand the biological effects driven by metabolites. In addition, the bias and missing information in their underlying databases pose inherent limitations on more focused analyses, such as on microbial tryptophan metabolism. To elucidate the microbial contributors for tryptophan metabolites, it is necessary to have a dedicated resource covering all the existing reactions in tryptophan degradation.

The rapid advancements in omics technologies and bioinformatics have joined forces to systematically decipher the function of gut microbiome. For instance, metagenomics and metabolomics led to the discovery of thousands of microbe-derived small molecules, as well as the genes associated with their productions [20]. Meanwhile, genome-scale metabolic models (GEMs) aim to accurately capture an organism’s metabolism by integrating information obtained from genome annotation, biochemical reaction, manual curation, and literature review. High-quality GEMs are now available for numerous microorganisms and serve as good resources for large-scale investigations of microbial tryptophan metabolism [21]. Several recent studies have employed GEMs to infer the relationships between phenotypic differentiation and metabolic capacities [22], to predict drug targets [23], and to understand tryptophan-metabolizing microbes involved in murine diarrhea [24]. The tryptophan metabolism pathways can be extracted from the GEMs to help understand the tryptophan metabolism distribution across the gut microbiome.

Here, we introduce TrpNet (https://www.trpnet.ca) which systematically collects the metabolites, reactions, and enzymes involved in tryptophan metabolism with specific attention to their microbial producers. TrpNet currently describes the tryptophan degradation pathways across ~5000 bacterial species including most known gut microbial species. Users can easily navigate TrpNet to find the origins of tryptophan metabolites or to generate tryptophan metabolism networks for selected microbes. Lastly, TrpNet allows researchers to predict tryptophan metabolites from any given taxonomic profiles on the basis of a Bayesian logistics regression model.

## 2. Results

**Figure 1:**
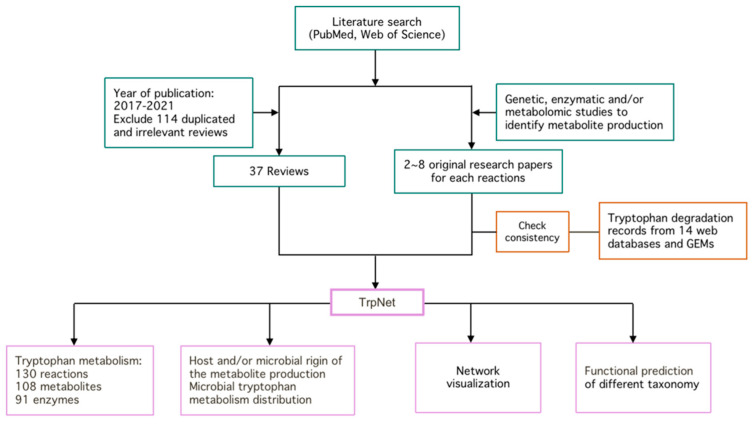
The development of TrpNet.

The overall workflow for the development of TrpNet is shown in Figure 1. The details of key steps are described in the subsequent sections.

### 2.1. Literature Search and Intestinal Tryptophan Metabolism

We define the tryptophan metabolism pathway as a set of reactions that transfer tryptophan to an end product without further searchable reactions or breakdown of tryptophan until the energy metabolism. To enumerate the metabolic reactions and metabolites regarding tryptophan biotransformation in microbes and their mammalian hosts, we manually searched and compared over 300 biochemical and metabolomic research papers on tryptophan metabolites, 37 reviews published in the last 5 years, and 14 public databases.

**Figure 2:**
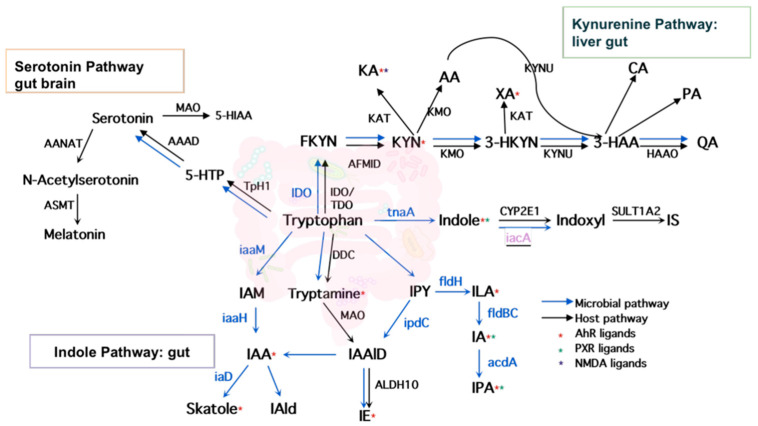
Host and microbial tryptophan metabolism pathways.

As shown in Figure 2, tryptophan metabolism generates 29 bioactive metabolites via three major pathways—indole pathway, serotonin pathway, and kynurenine pathway. The indole pathway, converting tryptophan into indole derivatives, including AhR ligands, predominates in gut microbes, while the serotonin and kynurenine pathways predominate in mammalian hosts. However, the origins of some tryptophan metabolites, whether microbe-derived or host-derived, are inconsistent across previously published reviews. In addition, most reports focused on the indirect roles of the gut microbiome in modulating kynurenine and serotonin production through non-tryptophan metabolites such as butyrate, an important SCFAs derived from gut microbes [25]. The direct production of kynurenines and serotonin by gut microbes is not well characterized. For instance, serotonin was termed as a host-limited metabolite [26], yet our investigation showed that several species such as *Lactococcus lactis*, *Lactobacillus plantarum*, and *Klebsiella pneumoniae* produced serotonin in a similar way to their mammalian host via aromatic amino-acid decarboxylase (AAAD) [27,28,29]. Another important neurotransmitter, tryptamine, was traditionally regarded as a microbial metabolite produced by *Clostridium*, *Ruminococcus*, *Blautia*, and *Lactobacillus* through tryptophan decarboxylases [13]. However, it is also reported to be produced by brain cells in certain cases and may play specific roles in the mammalian brain [30]. In addition, some gut microbes can degrade tryptophan in a different way through the kynurenine pathway. For instance, gut species *Burkholderia cepacia* was reported to convert tryptophan to 2-amino-3-carboxymuconate semialdehyde, which was further enzymatically degraded to pyruvate and acetate via the intermediates 2-aminomuconate and 4-oxalocrotonate rather than the known mammalian pathway which transforms 2-aminomuconate to 2-ketoadipate and, ultimately, glutaryl-coenzyme [31]. Compared with the most recent reviews [26,32,33,34] on the bioactive tryptophan metabolites, we updated the origin of all collected tryptophan metabolites including three inconsistent annotations of the kynurenines according to the current literature searches. The results were further cross-validated and enhanced with the information obtained from mining the GEMs, as described below.

### 2.2. Curation of Genome-Scale Metabolic Models

GEMs are knowledge-based stoichiometric-balanced metabolic networks containing the entire set of metabolic reactions, genes, and metabolites in the target organism [35]. Current developments in systems biology allow for the large-scale reconstruction of GEMs for numerous microorganisms. For instance, AGORA is a set of semiautomatically generated GEMs for 818 gut bacteria [36], and EMBL_GEMs is another large collection (5584 bacteria) for all reference and representative bacterial genomes of NCBI RefSeq [37] using CarveMe [38]. The reconstruction tools for both AGORA (assembly of gut organisms through reconstruction and analysis) and EMBL_GEMs were evaluated outstanding among the general tools, especially in gap-filling the network [39].

**Figure 3:**
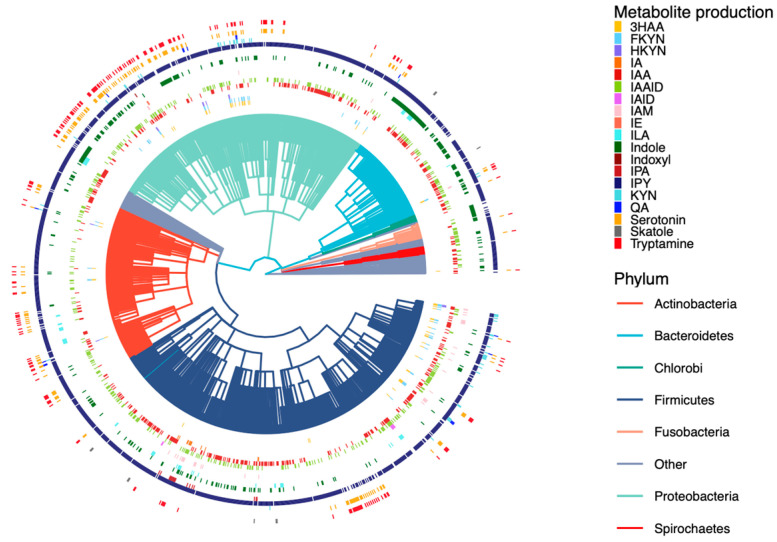
Distribution of tryptophan metabolite production across the human gut microbiome. Each branch indicates a microbe strain colored on the basis of their phyla (3HAA: 3-hydroxyanthranilate; FKYN: formylkynurenine; HKYN: 3-hydroxykynurenine; IA: 3-indoleacrylate; IAA: indole-3-acetate; IAAlD: indole-3-acetaldehyde; Iald: indole-3-carboxaldehyde; IAM: indole-3-acetamide; IE: indole-3-ethanol; ILA: indolelactate; IPA: indolepropionate; IPY: indolepyruvate; KYN: l-kynurenine; QA: quinolinate).

A total of 6402 GEMs covering 41 phyla were collected from AGORA and EMBL_GEMs. Most GEMs are at a strain level except for 73 at the species level and 333 models belonging to same strains shared between the two datasets. GEMs were manually annotated according to literature searches [40,41,42,43,44], of which 2114 models were labeled as the human gut microbe covering 1380 species of 30 phyla, and 177 were part of the mouse gut microbiome from 98 species of 10 phyla. The reactions, metabolites, and enzymes involved in microbial tryptophan metabolism were extracted from GEMs. These include nearly 5000 species belonging to 39 phyla and involve tryptophan metabolism covering 1246 species in the human gut and 88 species in the mouse gut (Figure 3). The results were corroborated by literature searches to reconcile inconsistencies between AGORA and EMBL_GEMs, as well as to make the GEM data as complete as possible.

### 2.3. Development of a Database for Tryptophan Metabolism and Functional Prediction

Following two major procedures described above, the final tryptophan metabolism pathway contains the entries for 130 reactions and 108 metabolites (excluding currency compounds such as water, hydrogen, oxygen, etc.) linking to 91 enzymes and more than 5000 GEMs. We developed a user-friendly web-based database and visual analytics tool - TrpNet (https://www.trpnet.ca/, accessed on 23 November 2021) to share this resource with the community. Users can browse, search, and filter reactions, metabolites, or microbes involved in tryptophan metabolism and visualize more detailed information and summary tables in multiple formats. Whenever possible, different entries are hyperlinked to PubMed, KEGG [14], BioCyc [15], and ModelSEED [21].

**Figure 4:**
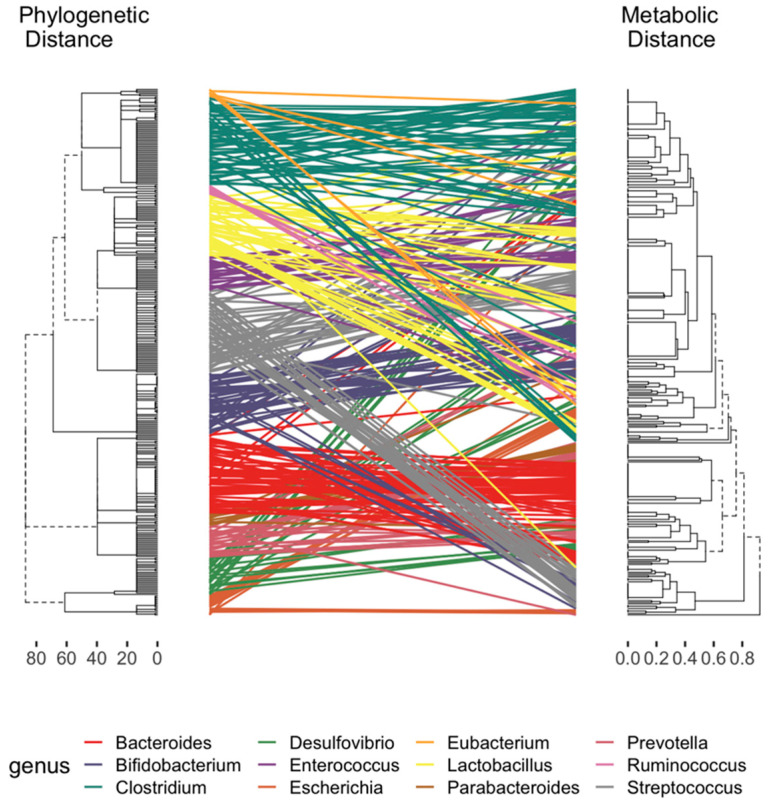
Tanglegram between the dendrograms of phylogenetic and metabolic distance. The phylogenetic dendrogram generated by hierarchical clustering with complete linkage of the taxonomy rank of maximum likelihood tree. The dendrogram of metabolic distance was calculated on the basis of the presence or absence of tryptophan metabolite production. Lines are colored by genus and connect the same microbes.

A main motivation of developing TrpNet is to help understand the relationship between the gut microbiome composition and the capacity for tryptophan metabolism. We designed the interface and functions to allow users to easily obtain the distribution of tryptophan metabolite production at different taxonomy levels. Figure 4 shows the pairwise distance between the phylogenetic tree from the dominant genus in the host gut and the corresponding metabolic clusters, according to the presence or absence of tryptophan metabolite production. It can be observed that phylogenetically close species may differ in their capacities in metabolite production. These data will help to resolve some inconsistencies between microbiome and metabolome divergence and the coexistence of specific species [22,45]. For instance, *Bacteroides* were found to be relatively conservative while *Lactobacillus* fluctuated in tryptophan metabolite production depending on whether they produced indole derivatives. Human- and mouse-specific gut microbes differed in the production of several AhR ligands such as IA, IAA, ILA, and IPA. This may help explain the different affinities of human AhR and mouse AhR in selecting exogenous ligands as reported in several studies [46,47] and shown in Figure S1.

Several computational tools such as PICRUSt2 [18] and Tax4Fun2 [19] are available for predicting functional profiles from 16S rRNA gene sequence data. Their performances are inherently limited by the known annotated enzyme groups which may not represent the metabolite generation. Specifically, public databases used in current tools are not tailored for tryptophan metabolism, and this may lead to bias due to incomplete information. TrpNet provides a more complete tryptophan metabolism according to literature curation and GEMs that describe metabolism at strain level with the potential to predict unknown enzymatic reactions. Here, we explored whether we could better predict the microbial tryptophan metabolism using the TrpNet database.

**Figure 5:**
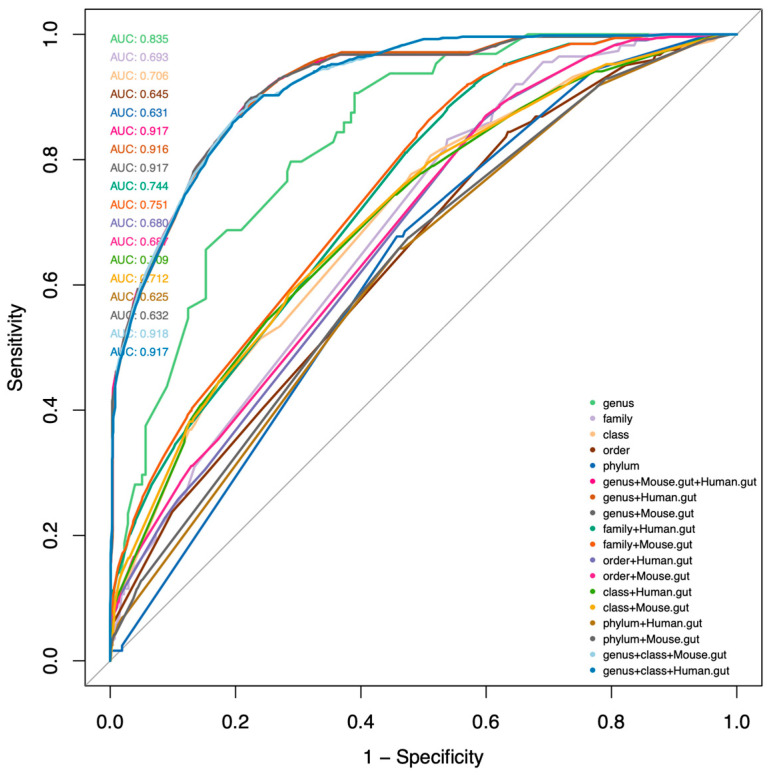
ROC plot for top-ranked IAA models based on prediction of stimulated data. The curves are colored by different models which use different predictors as listed for predicting IAA production.

One constraint is that 16S rRNA data cannot reach the resolution of strain level but usually identify the microbiome at the genus level. To address this issue, we used a logistic regression model to estimate the tryptophan metabolite production potential of the interested genus depending on the metabolite distribution collected by TrpNet. This approach was used in previous studies [48,49,50] to model microbiome compositional data and to identify informative microbiome features. To acquire more accurate models for our prediction, we fit Bayesian logistic regression models for each tryptophan metabolite according to their distributions across the taxonomy levels. In this model, the human/mouse gut origin was included as a nonrandom covariate as tryptophan metabolite production differs by the niche. Table 1 and Table 2 show the estimated odds ratios for the prevalence genus in producing bioactive indoles generated from mouse model and human model, respectively. The models were firstly validated by randomly split TrpNet database, whereby 80% was used for training and 20% was used to evaluate the model performance. We found that genus levels provided relatively reliable results for different metabolites in general. Figure 5 shows the ROC curves of the prediction models comparing different taxonomic levels in predicting IAA production. Please note that the performance measures are likely to be inflated as the same database was used for calculating the parameters of the regression models.

**Figure 6:**
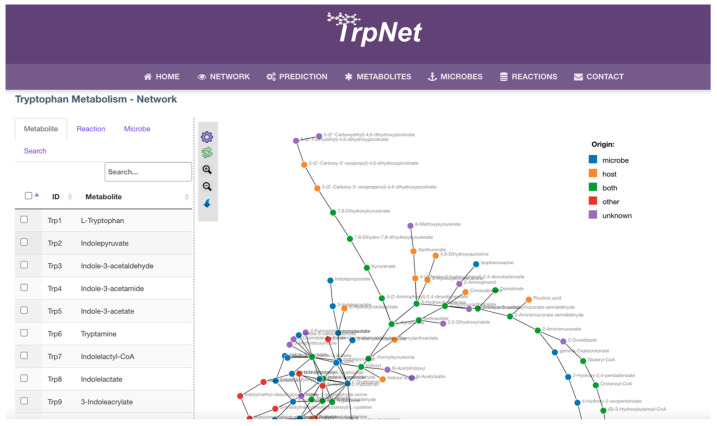
A screenshot of TrpNet showing the overall tryptophan metabolic network.

A network visualization page was implemented to allow users to search metabolites of interest in the network or to customize the tryptophan metabolism network according to a user-specified list of microbes (Figure 6). The result can be highlighted against the whole network or downloaded as a table. Another key feature of TrpNet lies in the annotation for the origin beyond the enzyme level. Reactions and metabolites were individually checked against the literature to label them as host-derived or microbial-derived, to help decipher the host–microbe interactions and co-metabolism.

### 2.4. Case Studies

#### 2.4.1. Myocardial Infarct (MI) Case Study

Disturbed tryptophan metabolism is known to alter the host inflammation status and affect many diseases including heart diseases such as myocardial infarction (MI) with an increased ratio of KYN/TRP [51]. To understand gut microbiome and host MI status with tryptophan metabolism, we collected 16 cecal samples from 16 mice (8 with MI and 8 control) day 3 post MI. Each sample was processed for 16S rRNA bacterial sequencing and untargeted metabolomics based on LC–MS and MS/MS. As it has been reported that females and males have differences in the risk of MI, we included data from male mice in our case study to exclude any additional effects of sex [52].

DADA2 [53] was used to assign taxonomy to amplicon sequence variants (ASVs). After filtering the 712 low-quality features, the remaining 304 ASVs were attributed to 69 genera dominated by Lachnospiraceae spp. And Ruminococcaceae spp. For metabolomics data, XCMS [54] and metID [55] were used for spectrum processing and peak annotation. A total of 24 microbial tryptophan metabolites were detected in LC–MS/MS, of which nine metabolites were significantly different including IAA, IAM, IalD, and serotonin. Statistical analyses of microbiome data were performed using MicrobiomeAnalyst [56]. Principal component analysis (PCA) evaluation showed that male mice without an MI differed from male mice post MI in microbiome composition. This was caused by a lack of Proteobacteria and Verrucomicrobia, which are active tryptophan metabolites producers in the no MI group (Figure S2).

**Figure 7:**
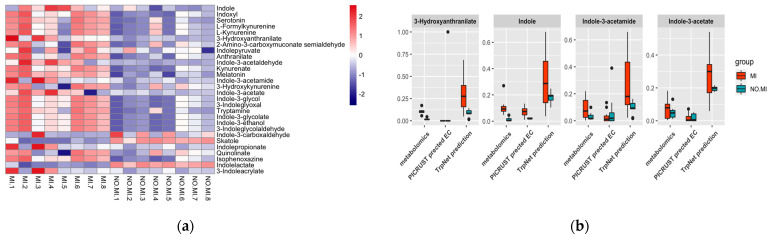
(**a**) Tryptophan metabolite prediction from microbiome data; (**b**) TrpNet prediction compared with metabolomics data and PICRUSt prediction.

Prediction models built on the TrpNet database were used to predict tryptophan metabolite production as a function of the genus-level data. Figure 7 shows the prediction result from the gut microbiome, as well as the comparison with metabolomics data and related enzymes predicted by PICRUSt2. Tryptophan degradation of the MI group differed significantly from their counterparts without MI, which may be explained by their diverse gut microbiome composition. According to our prediction, the MI group is more likely to produce greater amounts of tryptophan metabolites, including AhR ligands such as indole, IAM, and IAA, supporting the metabolomics data. Previous evidence has suggested that AhR activity is a critical modulator in the development and pathogenesis of the cardiovascular system [57]. AhR knockout mice were reported to be more susceptible to cardiac hypertrophy, vascular remodeling and systemic hypertension [58]. However, AhR activation can also contribute to the formation and promotion of atherosclerosis through inducing vascular inflammation [59]. Further studies are necessary in order to elucidate the effects on MI progression triggered by microbial AhR ligands from tryptophan metabolism.

Our prediction also shows increased activation of the kynurenine pathway post MI. This suggests that gut microbes may directly contribute to the increased KYN/TRP ratio, leading to the decreased level of beneficial serotonins and accumulation of neurotoxic KYN metabolites during the disease process. In parallel, we performed analyses using PICRUSt2. Only enzyme EC 4.1.99.1 relating to indole production was identified as significantly increased in the MI group, similar to the prediction by TrpNet and metabolomics data. Thus, TrpNet can serve as a better resource for exploring intestinal tryptophan metabolism.

#### 2.4.2. IBD Case Study

Previous studies have demonstrated the key role of the gut microbiome in IBD, and some highlighted the potential link to gut tryptophan metabolism [60]. The 16S rRNA and metabolomics data were collected from 26 participants between age 6 and 19 randomly selected from the Inflammatory Bowel Disease Multi-omics Database (http://ibdmdb.org, accessed on 17 October 2021) [61]. For each data type, 20 samples from pediatric Crohn’s disease (CD) patients and 20 from pediatric healthy controls were also included.

**Figure 8:**
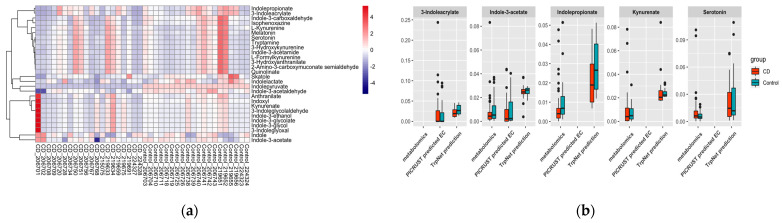
(**a**) Tryptophan metabolite prediction from microbiome data; (**b**) TrpNet prediction compared with metabolomics data and PICRUSt2 prediction.

From the metabolomics data annotation, nine tryptophan-derived metabolites were observed among which seven could be produced by the microbiome. IPA was significantly decreased in the CD group. Regarding the ASV sequencing data, 147 ASVs were annotated to 44 genera after filtering out the low-abundance features. However, there were no significant differences observed regarding microbiome composition between the CD and control group (Figure S3). TrpNet was then used for tryptophan metabolite prediction for each sample using the established model. Figure 8 shows the predicted distribution of tryptophan metabolites in comparison with metabolomics data and EC identified by PICRUSt2. Our prediction found the alteration of IPA validated by metabolomics and the obligatory intermediate IA in producing IPA from tryptophan. In contrast, PICRUSt2 did not contain the information for IPA, and the enzyme for IA was not significantly different between CD patients and healthy controls. Indole derivatives were predicted by TrpNet to be more abundant in healthy people than in CD patients, which is consistent with a previous report showing a reduction in AhR ligands by the microbiota in IBD patients. Most metabolites did not show significant differences between the two groups, probably due to the disperse microbiome structure. Interestingly, although indolepyruvate, which can improve intestinal epithelial barrier function during challenges with inflammatory stimuli, was not annotated by the metabolomics data, our prediction shows its decrease in CD patients, replicating previous results [60]. Despite serotonin being found increased in the CD group by metabolomics analysis, which was possibly due to the decreased expression of SERT in the ileum and colon [62], there was no significant difference according to our prediction. Similarly, the kynurenines were not different between the two groups using all the methods. Consequently, we can envision that gut microbes may affect IBD processing through tryptophan-derived AhR ligands such as IPA and IPY.

## 3. Discussion

Tryptophan metabolism plays a central role in host physiologic and pathologic processes. The balance among microbial tryptophan metabolism, supplementation, and microbial modulation exerts a great impact on local gastrointestinal and circulating tryptophan availability for its host and ultimately contributes to host health and disease. Hence, it is important to fully characterize tryptophan metabolism within a host or within its resident gut microbes. TrpNet, a first step toward addressing this gap, includes a collection of all currently known reactions and metabolites relating to tryptophan according to comprehensive literature reviews and large-scale data mining across >5000 GEMs. However, despite our intensive curation efforts, several reactions and metabolites are still left without related literature reports. For example, no reaction details are currently available for several tryptophan metabolites such as for Iald, an important AhR ligand.

One of the major challenges in microbiome studies is to determine the causal role that the gut microbiome composition plays in specific phenotypes. This is difficult due to the complexity of host–microbe interactions and microbe–microbe interactions. TrpNet can help decipher this co-metabolism by providing the detailed tryptophan metabolism within specific microbial species according to GEMs and literature annotation. Many current studies are based on 16S rRNA sequencing, making it essential to improve functional prediction and maximize the information gained from these relatively low-resolution taxonomic profiles. Here, we took an initial trial to predict tryptophan metabolism from genus-level bacterial identification using a logit regression model based on the TrpNet database. It should be noted that this prediction is limited by the current knowledge of the tryptophan metabolism, as well as algorithm for GEM construction or function prediction. Optimized methods are needed to improve the annotation of microbe to metabolite levels for mechanical and therapeutical insights. For instance, an increased KYN/Trp ratio has been reported as a potential biomarker for inflammation status, and supplementation of gut species that can naturally produce AhR ligands such as *Lactobacillus* spp. could help recover the AhR signaling. This microbe-based therapeutic approach was successfully applied in a mouse model of colitis [11]. As the gut microbiome can also modulate tryptophan metabolism indirectly by producing other small molecules such as bile acids, it will be useful to gather the information of microbes involved in these relevant processes to further improve TrpNet.

## 4. Materials and Methods

### 4.1. Literature Review

Review papers were searched from PubMed, Web of Science, and bioRxiv (www.biorxiv.org/, accessed on 8 October 2021) using the search term “tryptophan metabolism AND gut microbiome” since 2017. Those studies providing a global view of and tryptophan metabolism and focusing on the host–microbe interaction were included. Furthermore, for each tryptophan metabolite, research paper surveys were conducted to determine its origin. These papers showed showing at least one of the sources of genetic, enzymatic, or metabolic evidence in certain microbial species were prioritized.

### 4.2. GEMs Collection

A total of 818 GEMs in AGORA were collected from the Virtual Metabolic Human (VMH) database that can be accessed via the website (http://vmh.life, accessed on 3 September 2021), and EMBL GEMs were download from the EMBL BioModels website (https://www.ebi.ac.uk/biomodels, accessed on 3 September 2021). SBML files were parsed using R studio (version 4.1.1). GEMs were first annotated to human and/or mouse gut microbes on the basis of several large-scale studies and public gut microbiome databases. The models without records were then manually searched in PubMed to annotate their habitat.

### 4.3. TrpNet Implementation

The web-based database was developed on the basis of the JavaServer Faces (JSF) technology using the PrimeFaces framework (v11). The network visualization was implemented using D3 (version 5.0).

### 4.4. Sample Collection for MI Case Study

The murine experiments were approved by the Lady Davis Facility Animal Care Committee and followed the guidelines described by the Canadian Council on Animal Care. Retired breeder male mice were purchased from Charles River, St. Constant, PQ, Canada. Mice were housed in single cages on irradiated corn cob bedding in a vented rack, fed an irradiated Harlan Teklad Global 2018 diet which contains no animal protein and acidified tap water, and acclimated to the facility for 1 month before use. Surgery to create an MI was performed by the Surgery Core of the Lady Davis Institute [63,64]. Samples of cecal contents were collected day 3 post MI from a total of eight male mice, as well as from eight male mice which did not experience MI surgery. DNA for 16S rRNA sequencing was isolated using a Qiagen QIAamp PowerFecal DNA kit according to the manufacturer’s instructions. DNA samples were quantified, purity was determined, and samples sent to the McGill Genome Center. There, the bacterial V4 region was PCR-amplified from bases 515F to 806R, sequenced using a MiSeq Reagent Kit v3 (600-cycle), and run on an Illumina MiSeq. Data were processed and returned as amplified sequence variants (ASVs). R package DADA2 v 1.20.0 [53] was used to determine the abundance and gut bacterial species assignment. Gut metabolomics data from the same cecal contents were processed using an Orbitrap Q-Exactive LC–MS system in both positive and negative mode using a C18 column. MS/MS spectra were collected using data-independent acquisition (DIA). Raw LC–MS spectra were processed by MetaboAnalyst v5.0 [65] to generate a peak list table. About 150 MS1 peaks were found to be from potential tryptophan metabolites. According to MS/MS data, 24 tryptophan metabolites were identified using the metID package [52] (Table S1).

### 4.5. Sample Collection for IBD Case Study

The dataset of pediatric IBD stool samples was downloaded from the Integrative Human Microbiome Project Consortium (iHMP) [66]. For evaluation purposes, we randomly selected individuals between age 6 and 19 for disease (diagnosed with Crohn’s disease) and control groups. The information of the sample is listed in Table S2, and the original data can be found at https://ibdmdb.org/ (accessed on 17 October 2021). The tryptophan metabolites were extracted on the basis of annotation information provided by the authors (Table S3).

### 4.6. Logistic Regression Model for Predicting Metabolite Profiles

The logistic regression model was used to infer the metabolic profile from known taxonomy compositions. This method is from the generalized linear model family and can learn probabilistic models to predict the outcome of a binary variable from one or more response categorical or continuous variables. In our case, we aimed to predict tryptophan metabolite production using taxonomy profile and host type. The algorithm involved four key steps as described below.

1. Different taxonomy levels and their combinations were evaluated for their predictive values. Models were ranked by Akaike information criterion (AIC). The genus level combined with the host type was selected as the best predictor;
2. The models were further optimized by Bayesian logistic regression coupled with a fast Pareto smoothed leave-one-out cross-validation for the penalized likelihood estimation [67]. These models capture the metabolite production potential (PM, G) for the underlying metabolite (M) of interest in every genus (G) for a given host type;
3. The predicted probability (P_M,G_) was multiplied by the genus abundance table obtained from 16S rRNA sequencing data to compute the accumulated production potential for each metabolite of interest for each sample;
4. The results of all samples were normalized by total sum scaling to be comparable with metabolomics data.

## 5. Conclusions

Understanding molecular dialogues between the gut microbiome and the host is critical for developing microbiome-based diagnostic and therapeutic approaches. In this manuscript, we focused on improving our knowledge on tryptophan metabolism by integrating information from >5000 GEMs, 14 databases, and >300 literature reports. Through its user-friendly interface and interactive visualization, TrpNet provides the most up-to-date information for researchers to study tryptophan metabolism within the context of host and microbiome interactions. According to this information, we further developed an algorithm for predicting the microbial tryptophan metabolism from the 16S rRNA abundance profiles. Our two case studies demonstrated that our approach gives more accurate results compared to other established methods. We hope that TrpNet will be a useful resource that allows researchers to better understand the gut microbial tryptophan metabolism in the context of the gut microbiome for translational applications.

## Figures and Tables

**Table 1:** Odds ratio of dominate genera in mouse gut for bioactive indole generation (red: *p*-value < 0.001; orange: *p*-value < 0.01, yellow: *p*-value < 0.05, blue *p*-value < 0.1 based on Wald test).

Predictors	IA	Indole	IAAlD	IAM	IAA	ILA	IPA	Tryptamine
*Bacteroides*	0.8786	310.4118	1.5256	0.5621	2.9424	69.0048	0.8515	0.1484
*Bifidobacterium*	0.8712	0.0421	0.4879	0.6081	1.0597	103.1476	0.8393	8.5582
*Clostridium*	413.0681	2.2526	1.6328	106.6308	0.8401	89.0063	638.3164	3.473
*Desulfovibrio*	0.9738	1.5226	1.2451	0.8139	14.9215	0.8931	0.9676	0.3856
*Enterococcus*	0.9225	2.1667	0.8861	0.7017	0.0392	0.6446	0.9017	1.0872
*Escherichia*	0.936	241.1231	0.087	0.7997	3.9424	226.036	0.9161	9.0402
*Eubacterium*	0.9783	3.7121	0.622	0.8615	16.7253	0.8792	0.9723	0.4817
*Lactobacillus*	0.8536	0.0324	1.9794	0.5087	1.765	36.9424	0.8227	0.4338
*Mouse.gut*	2.5942	2.2931	1.1424	0.937	1.8319	6.9211	2.9119	0.4471
*Parabacteroides*	0.9668	0.1841	0.9344	0.8384	1.2605	13.8291	0.9569	0.4585
*Prevotella*	0.9145	1.6855	0.7029	0.5947	0.473	0.7286	0.8969	0.1589
*Ruminococcus*	0.9718	0.7449	0.4029	0.8035	7.2895	0.8861	0.9651	0.3699
*Streptococcus*	0.8959	0.6288	0.7287	0.5533	0.527	0.6756	0.8749	19.0245

**Table 2:** Odds ratio of dominate genera in human gut for bioactive indole generation (red: *p*-value < 0.001; orange: *p*-value < 0.01, yellow: *p*-value < 0.05, blue *p*-value < 0.1 based on Wald test).

Predictors	IA	Indole	IAAlD	IAM	IAA	ILA	IPA	Tryptamine
*Bacteroides*	0.8855	1595.5832	1.4595	0.5265	2.6489	79.5618	0.8575	0.1214
*Bifidobacterium*	0.9025	0.0371	0.7658	0.5674	0.7158	175.3057	0.8781	5.5164
*Clostridium*	414.0254	1.8606	1.2366	91.4966	1.6268	81.5643	606.5603	2.8663
*Desulfovibrio*	0.9683	1.55	0.6004	0.79	47.2592	0.8512	0.9593	0.3552
*Enterococcus*	0.9478	2.561	1.2673	0.7037	0.0322	0.7858	0.9333	0.9879
*Escherichia*	0.9639	318.856	0.081.	0.7677	4.0763	607.7244	0.9531	4.6206
*Eubacterium*	0.981	2.0208	1.2466	0.8559	10.5365	0.9035	0.9753	0.463
*Human.gut*	21.3204	1.4413	0.7778	342.7406	2.1685	20.4853	37.332	1876.3277
*Lactobacillus*	0.8757	0.1256	2.4492	0.5055	1.5343	52.7602	0.8462	0.412
*Parabacteroides*	0.976	0.1964	1.3015	0.8271	1.681	28.2969	0.9687	0.4121
*Prevotella*	0.9479	3.5273	2.0026	0.7035	1.294	0.7879	0.9332	0.2527
*Ruminococcus*	0.9663	0.6021	0.3477	0.7784	4.9819	0.8487	0.9562	0.3397
*Streptococcus*	0.8871	0.451	0.7435	0.5304	0.3467	0.6357	0.8596	18.3925

## Data Availability

The IBD data is available from http://ibdmdb.org (accessed on 23 November 2021). The MI data is available upon request.

## Supplementary Materials

The following are available online at https://www.mdpi.com/article/10.3390/metabo12010010/s1. Figure S1. Observed distribution of tryptophan metabolites production across the predominant genus in host gut. Microbial tryptophan metabolite production differs according to their host niche, Figure S2. Comparation of microbiome composition between MI and NO.MI group by (a) PCA plot and (b) bar plot, Figure S3. Comparation of microbiome composition between CD patients and health control by (a) PCA plot and (b) stack bar plot, Table S1. Metabolite annotation of MI case study, Table S2. IBD sample information, Table S3. Metabolite annotation of IBD case study.
