# Supplementary material for: TrpNet: Understanding Tryptophan Metabolism across Gut Microbiome
Source: Metabolites. 2021 Dec 23;12(1):10. doi: 10.3390/metabo12010010 (PMC8777792; doi:10.3390/metabo12010010)
Supplement: Supplementary file 1 [file metabolites-12-00010-s001.zip › metabolites-1500230-supplementary.pdf]

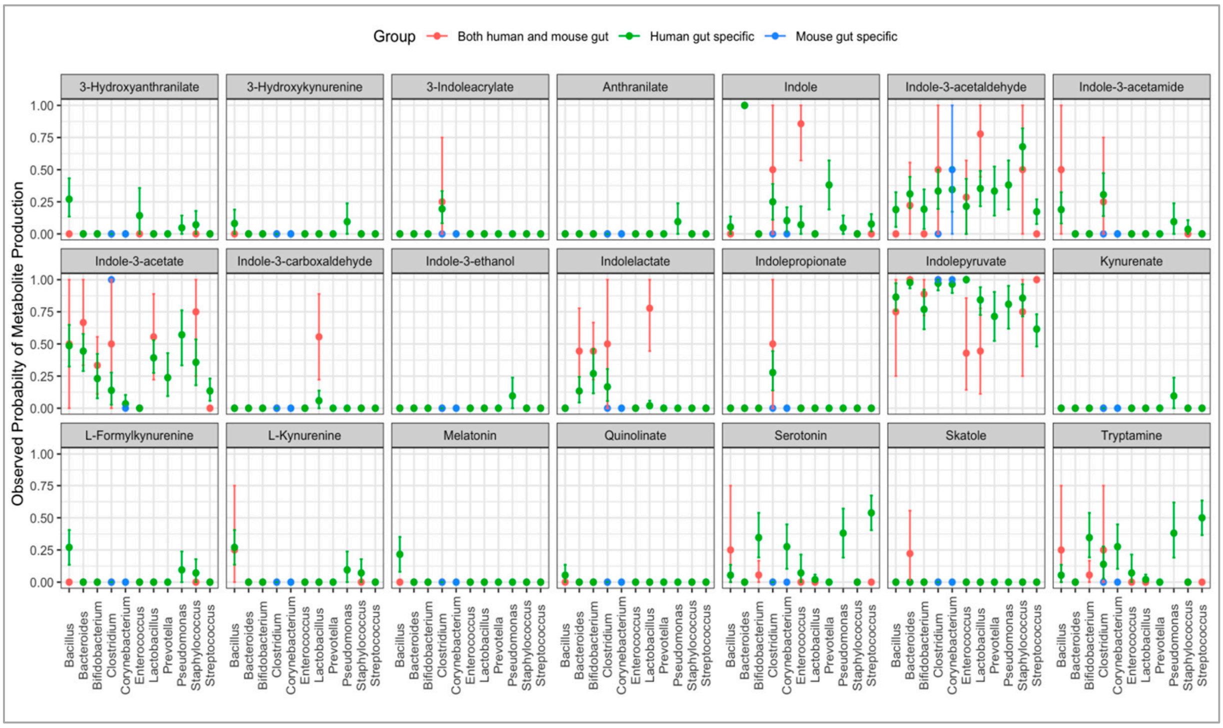

Figure S1. Observed distribution of tryptophan metabolites production across the predominant genus in host gut. Microbial tryptophan metabolite production differs according to their host niche.

a)

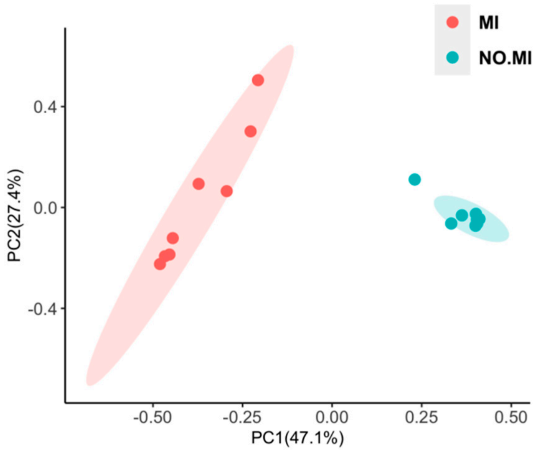

b)

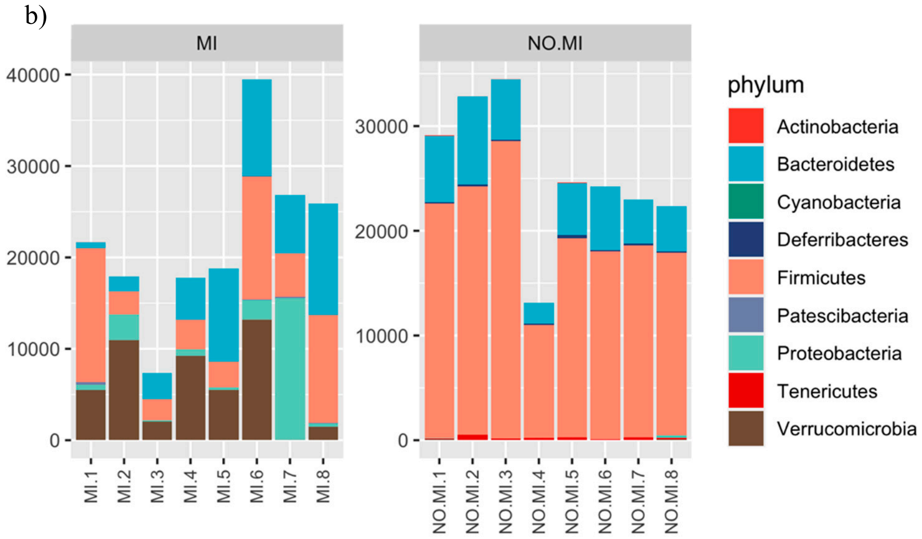

Figure S2: Comparation of microbiome composition between MI and NO.MI group by a) PCA plot and b) bar plot

a)

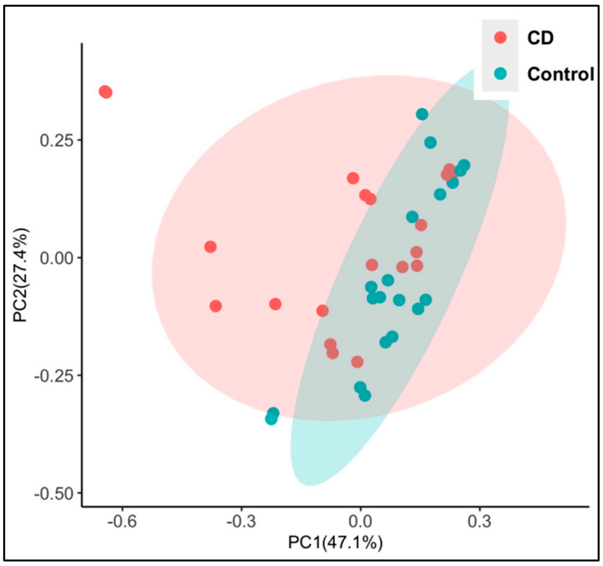

b)

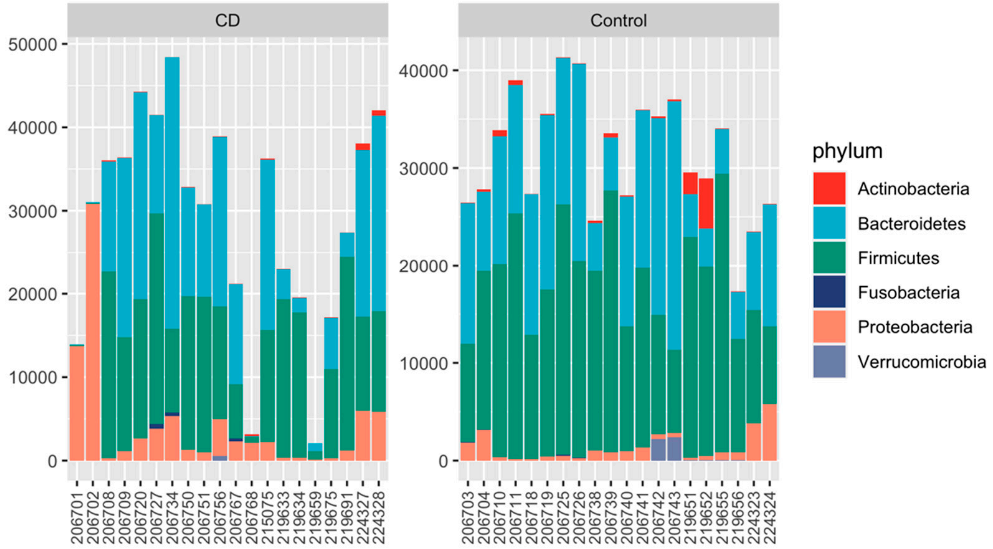

Figure S3 Comparation of microbiome composition between CD patients and health control by a) PCA plot, and b) stack bar plot.

Table S1: Metabolite annotation of MI case study

Method	m.z	r.t	metabolite	HMDB	p.val	q.val
C18-pos	118.0653	434.02	Indole	HMDB0000738	0.004849517	0.029097101
C18-pos	134.0601	50.05	Indoxyl	HMDB0004094	0.163702879	0.280633506
C18-pos	146.0601	193.95	Indole-3-carboxaldehyde	HMDB0029737	0.038698554	0.104664948
C18-pos	154.0498	75.19	3-Hydroxyanthranilate	HMDB0001476	6.23E-05	0.001494482
C18-pos	156.0655	42.47	2-Hydroxy-2,4-pentadienoate		0.677302058	0.738874972
C18-pos	175.0866	262.71	Indole-3-acetamide	HMDB0029739	0.009754728	0.046822692
C18-pos	177.074	299.03	Indole-3-acetate	HMDB0000197	0.405705958	0.512470684
C18-pos	205.0972	194.12	L-Tryptophan	HMDB0000929	0.201939133	0.323102614
C18-pos	233.1282	488.54	Melatonin	HMDB0001389	0.063520798	0.127041596
C18-pos	250.1183	534.17	L-Kynurenine	HMDB0000684	0.000761103	0.009133235
C18-pos	255.0974	345.01	L-Formylkynurenine	HMDB0060485	0.466500752	0.559800903
C18-pos	289.0793	47.4	5-Hydroxytryptophan	HMDB0000472	0.557691744	0.637361993
C18-pos	399.1441	42.72	S-Adenosyl-L-methionine	HMDB0001185	0.018157758	0.06225517
C18-neg	122.0235	52.38	Picolinic acid	HMDB0002243	0.733505941	0.765397504
C18-neg	131.0702	257.78	Acetaldehyde	HMDB0000990	0.051555973	0.112485759
C18-neg	182.0452	52.56	Anthranilate	HMDB0001123	0.329035844	0.438714458
C18-neg	188.0346	224.54	Kynurenate	HMDB0000715	0.002747476	0.021979806
C18-neg	196.0623	944.87	Serotonin	HMDB0000259	0.300856814	0.424739031
C18-neg	206.0455	231	4-(2-Aminophenyl)-2,4-dioxobutanoate	HMDB0000978	0.050268969	0.112485759
C18-neg	223.0735	808.61	3-Hydroxykynurenine	HMDB0011631	0.01656781	0.06225517
C18-neg	379.2125	350.32	N-Methylserotonin	HMDB0004369	0.074568093	0.137664172
C18-neg	650.2153	44.04	3-Methylindolepyruvate		0.225813862	0.338720792
C18-neg	223.0735	954.24	5-Hydroxykynurenine	HMDB0012819	0.039249355	0.104664948
C18-neg	131.0338	140.94	4-Hydroxy-2-oxopentanoate	HMDB0040531	0.921325626	0.921325626

Table S2: IBD sample information

Sample	Group	Participant.ID	Data
206701	CD	H4006	16s
206702	CD	H4006	16s
206703	Control	H4008	16s
206704	Control	H4008	16s
206708	CD	H4007	16s
206709	CD	H4007	16s
206710	Control	H4009	16s
206711	Control	H4009	16s
206718	Control	H4013	16s
206719	Control	H4013	16s
206720	CD	H4014	16s
206725	Control	H4016	16s
206726	Control	H4016	16s
206728	CD	H4017	16s
206734	CD	H4020	16s
206738	Control	H4022	16s
206739	Control	H4022	16s
206740	Control	H4023	16s
206741	Control	H4023	16s
206742	Control	H4024	16s
206743	Control	H4024	16s
206750	CD	H4028	16s
206751	CD	H4028	16s
206756	CD	H4031	16s
206767	CD	H4038	16s
206768	CD	H4039	16s
215075	CD	M2068	16s
219633	CD	P6005	16s
219634	CD	P6005	16s
219651	Control	P6014	16s
219652	Control	P6014	16s
219655	Control	P6017	16s
219656	Control	P6017	16s
219659	CD	P6016	16s
219675	CD	P6028	16s
219691	CD	P6033	16s
224323	Control	H4045	16s
224324	Control	H4045	16s
224327	CD	H4043	16s
224328	CD	H4043	16s

Sample	Group	Participant.ID	Data
HSM5MD4A	CD	H4006	metabolomics
HSM5MD86	CD	H4006	metabolomics
HSM5MD4A	CD	H4006	metabolomics
HSM5MD4Q	CD	H4007	metabolomics
HSM5MD7Z	CD	H4007	metabolomics
HSM5MD8A	Control	H4008	metabolomics
HSM6XRSZ	Control	H4008	metabolomics
HSM5MD82	Control	H4009	metabolomics
HSM67VGA	Control	H4009	metabolomics
HSM67VGG	Control	H4009	metabolomics
HSM5MD8H	Control	H4013	metabolomics
HSM6XRVK	Control	H4013	metabolomics
HSM5MD3L	CD	H4014	metabolomics
HSM6XRVO	CD	H4014	metabolomics
HSM5MD5Z	Control	H4016	metabolomics
HSM67VHM	Control	H4016	metabolomics
HSM7J4I3	Control	H4016	metabolomics
HSM6XRQB	CD	H4017	metabolomics
HSM7CYY3	CD	H4017	metabolomics
HSM67VCZ	CD	H4020	metabolomics
HSM67VHF	CD	H4020	metabolomics
HSM7J4PW	CD	H4020	metabolomics
HSM67VDX	Control	H4022	metabolomics
HSM7CYZJ	Control	H4022	metabolomics
HSM67VDR	Control	H4023	metabolomics
HSM67VI5	Control	H4024	metabolomics
HSM7J4MY	CD	H4028	metabolomics
HSM7J4N4	CD	H4028	metabolomics
HSM7CZ3G	CD	H4031	metabolomics
HSM7J4J7	CD	H4038	metabolomics
HSM7J4LP	CD	H4039	metabolomics
HSM7J4JW	CD	H4043	metabolomics
HSMA33NY	Control	H4045	metabolomics
MSM79HDQ	CD	M2068	metabolomics
PSM6XBQM	CD	P6005	metabolomics
PSM7J1DH	Control	P6014	metabolomics
PSM7J19B	CD	P6016	metabolomics
PSM7J19V	Control	P6017	metabolomics
PSM7J17F	CD	P6028	metabolomics
PSM7J188	CD	P6033	metabolomics

Table S3: Metabolite annotation of IBD case study

Method	m.z	r.t	metabolite	HMDB	p.val	q.val
C18-neg	174.0552	3.81	Indole-3-acetate	HMDB0000197	0.163984658	0.218646211
HILIC-neg	188.0718	4.36	Indolepropionate	HMDB0002302	0.032552121	0.062347944
HILIC-neg	212.0023	3.8	Indoxyl sulfate	HMDB0000682		
HILIC-neg	203.0828	3.27	L-Tryptophan	HMDB0000929	0.038967465	0.062347944
HILIC-neg	204.0302	8.3	Xanthurenate	HMDB0000881	0.458322297	0.458322297
HILIC-pos	205.0976	6.45	L-Tryptophan	HMDB0000929	0.021758252	0.062347944
HILIC-pos	221.0925	6.53	5-Hydroxytryptophan	HMDB0000472	0.012812667	0.062347944
HILIC-pos	177.1025	6.45	Serotonin	HMDB0000259	0.029886537	0.062347944
HILIC-pos	190.0503	5.16	Kynurenate	HMDB0000715	0.385805746	0.440920853
HILIC-pos	399.145	10.17	S-Adenosyl-L-methionine	HMDB0001185		
